# Population-genomic analyses reveal bottlenecks and asymmetric introgression from Persian into iron walnut during domestication

**DOI:** 10.1186/s13059-022-02720-z

**Published:** 2022-07-04

**Authors:** Ya-Mei Ding, Yu Cao, Wei-Ping Zhang, Jun Chen, Jie Liu, Pan Li, Susanne S. Renner, Da-Yong Zhang, Wei-Ning Bai

**Affiliations:** 1 State Key Laboratory of Earth Surface Processes and Resource Ecology and Ministry of Education Key Laboratory for Biodiversity Science and Ecological Engineering, College of Life Sciences, Beijing Normal University, Beijing, 100875 China; 2 China National Botanical Garden, Beijing, 100093 China; 3 CAS Key Laboratory for Plant Diversity and Biogeography of East Asia, Kunming Institute of Botany, Chinese Academy of Sciences, Kunming, 650201 Yunnan China; 4 The Key Laboratory of Conservation Biology for Endangered Wildlife of the Ministry of Education, College of Life Sciences, Zhejiang University, Hangzhou, 310058 China; 5 Department of Biology, Washington University, Saint Louis, MO 63130 USA

**Keywords:** Domestication bottleneck, Introgression, Iron walnut, Persian walnut, Shell-thickness gene

## Abstract

**Background:**

Persian walnut, *Juglans regia*, occurs naturally from Greece to western China, while its closest relative, the iron walnut, *Juglans sigillata*, is endemic in southwest China; both species are cultivated for their nuts and wood. Here, we infer their demographic histories and the time and direction of possible hybridization and introgression between them.

**Results:**

We use whole-genome resequencing data, different population-genetic approaches (PSMC and GONE), and isolation-with-migration models (IMa3) on individuals from Europe, Iran, Kazakhstan, Pakistan, and China. IMa3 analyses indicate that the two species diverged from each other by 0.85 million years ago, with unidirectional gene flow from eastern *J. regia* and its ancestor into *J. sigillata*, including the shell-thickness gene. Within *J. regia*, a western group, located from Europe to Iran, and an eastern group with individuals from northern China, experienced dramatically declining population sizes about 80 generations ago (roughly 2400 to 4000 years), followed by an expansion at about 40 generations, while *J. sigillata* had a constant population size from about 100 to 20 generations ago, followed by a rapid decline.

**Conclusions:**

Both *J. regia* and *J. sigillata* appear to have suffered sudden population declines during their domestication, suggesting that the bottleneck scenario of plant domestication may well apply in at least some perennial crop species. Introgression from introduced *J. regia* appears to have played a role in the domestication of *J. sigillata.*

**Supplementary Information:**

The online version contains supplementary material available at 10.1186/s13059-022-02720-z.

## Background

The gradual transition from hunting and gathering to plant cultivation and animal husbandry began between the end of the Pleistocene and the beginning of the Holocene, some 12,000–10,000 years ago [1]. Domestication of plants involves humans acting as dispersers and modifiers of a crop’s biotic and abiotic environment [2, 3]. It is a gradual process and often is not restricted to a single place or single human population [4]. Purposeful cultivation by humans reduces genetic diversity through so-called domestication bottlenecks [5–9] and simultaneously increases the frequency of domestication alleles [10]. As a result of domestication, crop plants share suites of modified traits, referred to as the domestication syndrome, that set them apart from their wild ancestors. These traits typically include reduction of physical and chemical defences in the parts used by humans, larger or more easily accessible fruits or seeds, and traits related to sugar or fat content. In long-lived tree fruit crops, such as pears, grapes, almonds, apples, peaches, or apricots, trait changes due to human selection in some cases appear to have taken a long time, in others instead seem to have been rapid and driven by hybridization [11–18]. The time and duration of initial domestication, and the possible role of introgression, can be inferred with multidisciplinary research, combining data from archaeology and population genomics. Such studies can pinpoint the geographic and ecological origin of crop plants, the inheritance of domestication traits, and the timing and speed of domestication [3, 10].

Inferring a geographic and temporal framework for the domestication process of a species requires broad geographic sampling, developing and testing models of migration and gene flow, and inferring changes in effective population size (*N*_*e*_) in the recent past, thereby proceeding from “storytelling to story testing” [19]. One reason why the bottleneck scenario has been challenged is that population-genomic analyses that rely on coalescent models, such as the pairwise (PSMC) or multiple sequential Markovian coalescent (MSMC) [20, 21], often reveal a gradual decline in *N*_e_ associated with domestication [10]. These methods have limited power over timespans within a few hundred generations [22], the timeframe during which the domestication of trees, with generation times of 30 or 50 years, must have occurred. A new method developed by Santiago et al. [23], called GONE, however, is able to infer the demographic history of a population within the past 100 generations from the observed spectrum of linkage disequilibrium (LD) of pairs of loci over a wide range of recombination rates in a sample of contemporary individuals. A particular advantage of the approach is its high tolerance for deviations from the assumptions of the model including population admixture and subdivision [23].

Among the world’s high-value tree crops is the Persian walnut, *Juglans regia*, cultivated for its fat-rich nuts and valuable wood. The nuts of both wild and cultivated Persian walnut are gathered and consumed [24, 25], but wild trees produce smaller fruits (2–3 cm in diameter) with thicker shells, while domesticated varieties have larger fruits (3–6 cm in diameter) with thinner shells [26]. A genomic study of Iranian walnuts was able to link a SNP on chromosome 13 to shell thickness [27], and two other studies have reported candidate genes for shell thickness [28, 29]. These inferences became possible with the release of the first walnut reference genome, Chandler v1.0 [30], which allowed whole-genome resequencing [29, 31, 32], the development of high-density genotyping tools [33], and the genetic dissection of agronomical traits in walnut, including the mentioned shell thickness [27, 28, 34–36]. Chromosome-level assemblies are now available for both *J. regia* and *J. sigillata*, the latter a close relative endemic in Guizhou, Sichuan, Southeast Xizang (Tibet), Yunnan, Bhutan, and Sikkim where its wood and nuts are used similarly to those of Persian walnut [37].

During the last glacial period, the genus *Juglans* went extinct over much of Europe, except for refugia near the Black Sea, in the southern Caspian region, the balkans, and Spain [25, 38]. After the retreat of the ice, *J. regia* (the only species native in Europe) reexpanded, perhaps from refugia in northern Turkey towards Greece, where walnut pollen becomes abundant by 3100–3300 BP [38–40]. Archaeological finds of nutshells and linguistic data suggest that walnuts were first domesticated in the Irano-Anatolian region, a biogeographic unit comprising parts of Iran, Turkey, the southern Caucasus, and Turkmenistan, and then was spread westward and eastward by humans [24, 25, 38, 41, 42]. The early use of walnuts in this region is clear from nutshells found inside a ceramic container in an Armenian grave site (southern Caucasus) associated with C-14-dated remains from 4230 to 3790 BC (~6200 years ago, Late Neolithic) [43]. The Persian walnut’s anthropogenic expansion eastwards could thus have occurred already during the early Bronze Age (5300–3100 years ago) as indicated by nutshells found in Kashmir near C-14-dated wheat and barley grains from 2700 to 2000 BC (~4700–4000 years ago) [44] and in northern Pakistan in soil below a market, with other C-14-dated remains from 1200 BC (~3200 years ago) [45]. The fossils’ presence below a former market suggests that these walnuts were actively collected and traded.

Alternatively, the eastward expansion of cultivated walnuts could have occurred via the Silk Road, which fully connected the Roman Empire to China from 90 to 130 AD onwards, when the Han Dynasty officially opened trade with the West [46]. In line with this hypothesis, Beer et al. [42] found that the walnut forests of Kyrgyzstan are at most 2000 years old, with most only about 1000 years old.

Here we analyze whole-genomic sequences from numerous individuals of *J. regia* from Europe, Iran, Kazakhstan, Pakistan, and China, as well as Chinese *J. sigillata*, to infer the demographics and timeframe for the spread of walnuts using both the PSMC [20] and GONE approach [23]. In other long-lived crops, including apples, pears, peaches, grapes, and apricots [11, 13–16, 18], hybridization has played a role in domestication, and we therefore thought it important to include *J. sigillata*, which hybridizes freely with *J. regia*, and to also infer this species’ demography, cultivation history, and possible introgression of traits relevant for domestication, such as shell thickness.

## Results

### Population structure and phylogenetic analyses

**Fig. 1:**
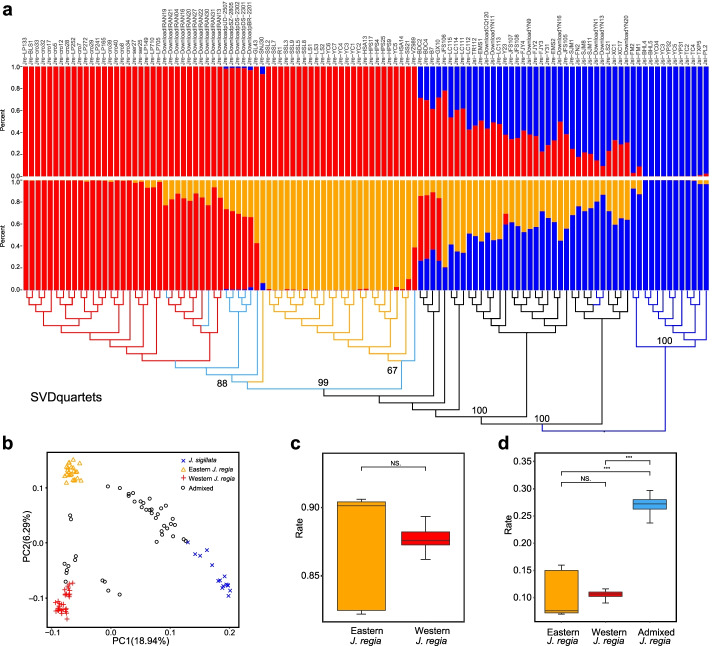
Population structure and phylogenetic analysis. **a** Population structure and phylogeny of 113 individuals of *Juglans regia* and *J. sigillata* from throughout both species’ ranges. Individuals in the structure plot are ordered according to their phylogenetic proximity. Red branches and bars represent the western group of *J. regia*, orange branches and bars the eastern group, blue branches and bars *J. sigillata*, black branches represent hybrids between eastern *J. regia* and *J. sigillat*a, and cyan branches hybrids between western and eastern *J. regia*. Values at nodes represent bootstrap support. **b** A principal component analysis (PCA) of 113 individuals of *J. regia* and *J. sigillata*. Blue crosses represent *J. sigillata*, orange triangles eastern *J. regia*, red pluses western *J. regia*, and black circles admixed individuals. **c** Proportions of SNPs in the western or eastern group of *J. regia* shared with the nine admixed individuals*.***d** Proportions of private SNPs in nine individuals of eastern, western, and admixed *J. regia*. The boxplots indicate the minimum (the lower hinge), maximum (the upper hinge), and median (the middle hinge). NS. means no significant difference, “***” *p* < 0.001, “**” *p* < 0.01, “*” *p* < 0.05

Based on the parsimony method [47] and a dataset of 2352 SNPs that are independent and non-coding (Methods) from 87 individuals of *J. regia* and 26 individuals of *J. sigillata*, the optimal number of populations in a STRUCTURE analysis was *K* = 3, while the delta *K* method suggested *K* = 2 as optimal (Additional file 1). At *K* = 2, one cluster consisted of the 65 individuals of *J. regia* from Europe (Belgium, Austria, Germany, Croatia, Serbia, Moscow), Western Asia (Iran), Central Asia (Kazakhstan), South Asia (Pakistan), and China, the other of the 16 *J. sigillata* from Southwest China (posterior probability >0.80; Fig. 1a), with 22 individuals of *J. regia* and 10 of *J. sigillata* exhibiting admixture. At *K* = 3, *J. regia* divides into an eastern and a western group, the former comprising the 25 individuals from China, the latter the 23 individuals from Europe and eight from Iran. A few individuals from Pakistan (five), Iran (two), Kazakhstan (one), and China (one from Xinjiang) showed admixture between the eastern and western group of *J. regia*, and 18 individuals of *J. regia* and 11 of *J. sigillata*, all from China, showed admixture between these two species as did four individuals of *J. regia* from Tibet (Fig. 1a, Additional file 2).

Results from a principal component analysis (PCA) of the 2352 SNPs were consistent with the STRUCTURE results, with the first two components explaining 18.94% and 6.29% of the total variance (Fig. 1b). All 113 individuals could be assigned to three groups (*J. regia*, *J. sigillata*, admixed) on PC1 and PC2; *J. regia* is separated into two groups along PC2.

We next reconstructed a phylogenetic tree from 22,048 genome-wide independent SNPs (Methods). The eastern and western groups of *J. regia* inferred in the STRUCTURE and PCA analyses formed separate clades (Fig. 1a), while two admixed individuals from Iran, five admixed individuals from South Asia (Pakistan), and one admixed individual from China (Xinjiang in northwest-most China) formed a basal grade in the western *J. regia* clade. One admixed individual from Kazakhstan fell near the base of the eastern *J. regia* clade (Fig. 1a). We also calculated the shared and private SNPs for the western and eastern group, as well as for the nine admixed individuals of *J. regia.* To eliminate the effect of unequal sample size, we randomly selected nine individuals from each group and repeated the calculation 20 times; 86.2 to 89.3% of the variation (SNPs) in the western group and 82.2 to 90.6% in the eastern group were shared with the admixed individuals (Fig. 1c). The latter had 23.7 to 29.7% private SNPs, many more than either the western or eastern group of *J. regia,* which had 9.0 to 11.6% and 7.0 to 16.0% private SNPs, respectively (Fig. 1d).

That the nine admixed individuals from Pakistan, Iran, Kazakhstan, and Xinjiang had more private SNPs and that eight of them formed a grade at the base of western *J. regia* clade (Fig. 1a) supports the hypothesis that two groups of *J. regia* diverged from each other in western Central Asia.

### Demographic history and inference of bottlenecks

**Fig. 2:**
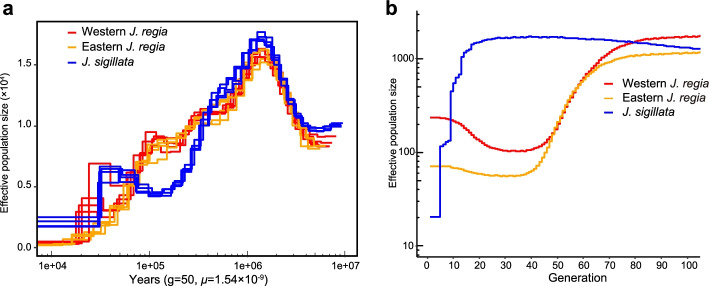
Population-demographic history of *Juglans sigillata*, and eastern and western *J. regia*. **a** Inferred with PSMC. **b** Inferred with GONE (Methods)

Using pairwise sequential Markovian coalescence, PSMC [20], we inferred the demographic histories of four individuals representing each of the three groups found in the STRUCTURE analysis. Western and eastern *J. regia* genomes yielded similar inferred demographic histories, with a high *N*_e_ at ~1.5 Ma followed by a decline at ~0.01 Ma (Fig. 2a). The demographic history of *J. sigillata* reflects a different trajectory since 0.5–0.6 Ma, with a more rapid decline than either of the *J. regia* groups at about ~0.4 Ma, a slight increase in *N*_e_ between 0.07 and 0.03 Ma, and a second rapid decline around 0.03 Ma (Fig. 2a). The demographic trajectories of the three groups are consistent regardless of which generation time, 30 or 50 years, is assumed (Fig. 2a, Additional file 3: Fig. S1).

As an alternative to the PSMC approach for inferring the demographics of *J. regia* and *J. sigillata*, we used GONE [23], with the maximum recombination rates set to 0.01 to reduce potential bias from recent migrants from another population (Methods). The results suggest that the western and eastern *J. regia* groups experienced a bottleneck between ~80 and 40 generations ago and an expansion at ~40 generations, while *J. sigillata* had a constant effective population size ~100 to ~20 generations ago, followed by a rapid decline (Fig. 2b). Assuming a generation time of 50 years, the bottlenecks in *J. regia* and *J. sigillata* would have occurred ~4000 and ~1000 years ago, respectively, while with an assumed generation time of 30 years, they would have occurred ~2400 and ~600 years ago, respectively.

### Divergence time and gene flow between *J. regia* and *J. sigillata*

**Fig. 3:**
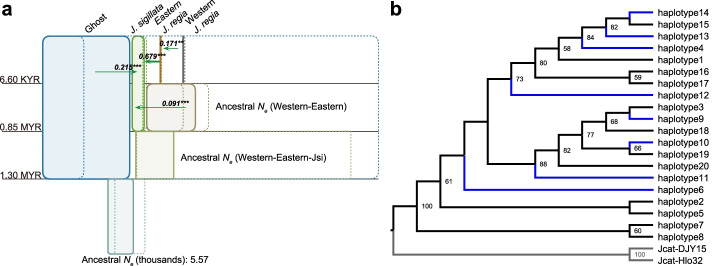
Divergence time and gene flow estimated for *Juglans regia* and *J. sigillata* with an IMa3 model that included a ghost population (Methods), and a phylogeny of shell-thickness gene sequences. **a** Each group is represented by a box of a width proportional to its estimated effective population size (ancestral *N*_e_ is given for scale). Confidence intervals are indicated as dashed-line boxes aligned with the corresponding population’s box on the left side. Green arrows represent an effective number of migrant gene copies per generation (*2Nm*) from the source population to the receiving population. Only statistically significant migration rates are shown (*P < 0.05; **P < 0.01; ***P < 0.001). **b** Maximum likelihood phylogeny obtained from 20 haplotypes of the shell-thickness gene from *J. regia* and *J. sigillata*. The blue lines represent 8 haplotypes of *J. regia* that are shared with *J. sigillata*. Ultrafast bootstrap (UFBoot) support values ≥ 50 shown at nodes

We estimated parameter values in several runs of non-ghost and ghost models, using Isolation-with-migration models (IMa3) [48] and assuming generation times of either 50 or 30 years (Additional file 3: Fig. S2 and Additional files 4 and 5). The ghost model assumed that the common ancestor of western and eastern *J. regia* first coalesced with *J. sigillata*, and then with the ghost population: (((western *J. regia*, eastern *J. regia*), *J. sigillata*), ghost population). The non-ghost model assumed that the common ancestor of western and eastern *J. regia* coalesced with *J. sigillata*: ((western *J. regia*, eastern *J. regia*), *J. sigillata*). The logs of marginal likelihoods under the ghost model were larger than those of the non-ghost model (mean marginal likelihood of – 185,498.034 and – 185,522.246 for the ghost model, − 185,592.761 and – 185,589.530 for the non-ghost model; Methods). Under the ghost model, western and eastern *J. regia* diverged from each other about 6636 years ago (95% HPD 2639–15,963 years) and *J. regia* and *J. sigillata* about 0.85 Ma ago (95% HPD 0.62–1.36 Ma). The divergence time of the common ancestor of *J. regia* and *J. sigillata* from the ghost lineage was estimated as 1.35 Ma (95% HPD 1.07–2.15 Ma) (Fig. 3a). The effective population size was 160 (95% HPD 75–362) for western *J. regia*, 111 (95% HPD 55–249) for eastern *J. regia*, and 2693 (95% HPD 2288–3154) for *J. sigillata* (Fig. 3a). The migration rate (2*Nm*) from the western to the eastern group of *J. regia* was 0.17, with a high rate of asymmetrical migration from eastern *J. regia* into *J. sigillata* (2*Nm* = 0.68). The migration rate from the ghost population to *J. sigillata* was 0.22 (Additional file 4).

### Adaptive introgression into *J. sigillata*

Since the IMa3 result supported the ghost model, we used Sprime [49] to detect the segments of ghost introgression in *J. sigillata* with the information of the genetic distance in centimorgans for each SNP and *J. regia* as the outgroup. We identified 19 genome segments (the average length was 156 kb) distributed in 10 chromosomes and involving 102 genes as part of ghost introgression into *J. sigillata* when assuming a generation time of 50 years (Additional file 3: Fig. S3). We used five statistics from three methods to identify 1195 genes under positive selection in *J. sigillata* at the genome-wide level (Methods). Overlapping the 102 genes of the ghost introgression with the 1195 genes under positive selection revealed nine genes with signals of adaptive introgression, of which six are involved in biotic and abiotic stress responses (Additional file 6: Table S5).

### A phylogeny of shell-thickness gene sequences

We used reciprocal best hits between protein sequences of the “Chandler” v2.0 [50] and “JrSerr” [51] walnut genomes to obtain the shell-thickness gene in “JrSerr” and extracted homologous shell-thickness gene from the consensus genomes of *J. sigillata* and *J. regia*. We identified a total of 20 haplotypes and then inferred a phylogeny of the shell-thickness gene. No haplotype was specific to *J. sigillata*, and eight haplotypes residing in that species were shared with at least one pure or admixed individual of *J. regia* from China (Fig. 3b; Additional file 7: Table S6). Altogether, these results indicate that the shell-thickness gene of *J. sigillata* was likely acquired from eastern *J. regia* by introgression. The shell-thickness gene tree was consistent with the IMa3 result (above) in suggesting unidirectional gene flow from eastern *J. regia* into *J. sigillata*.

### Chloroplast phylogenetic analysis

**Fig. 4:**
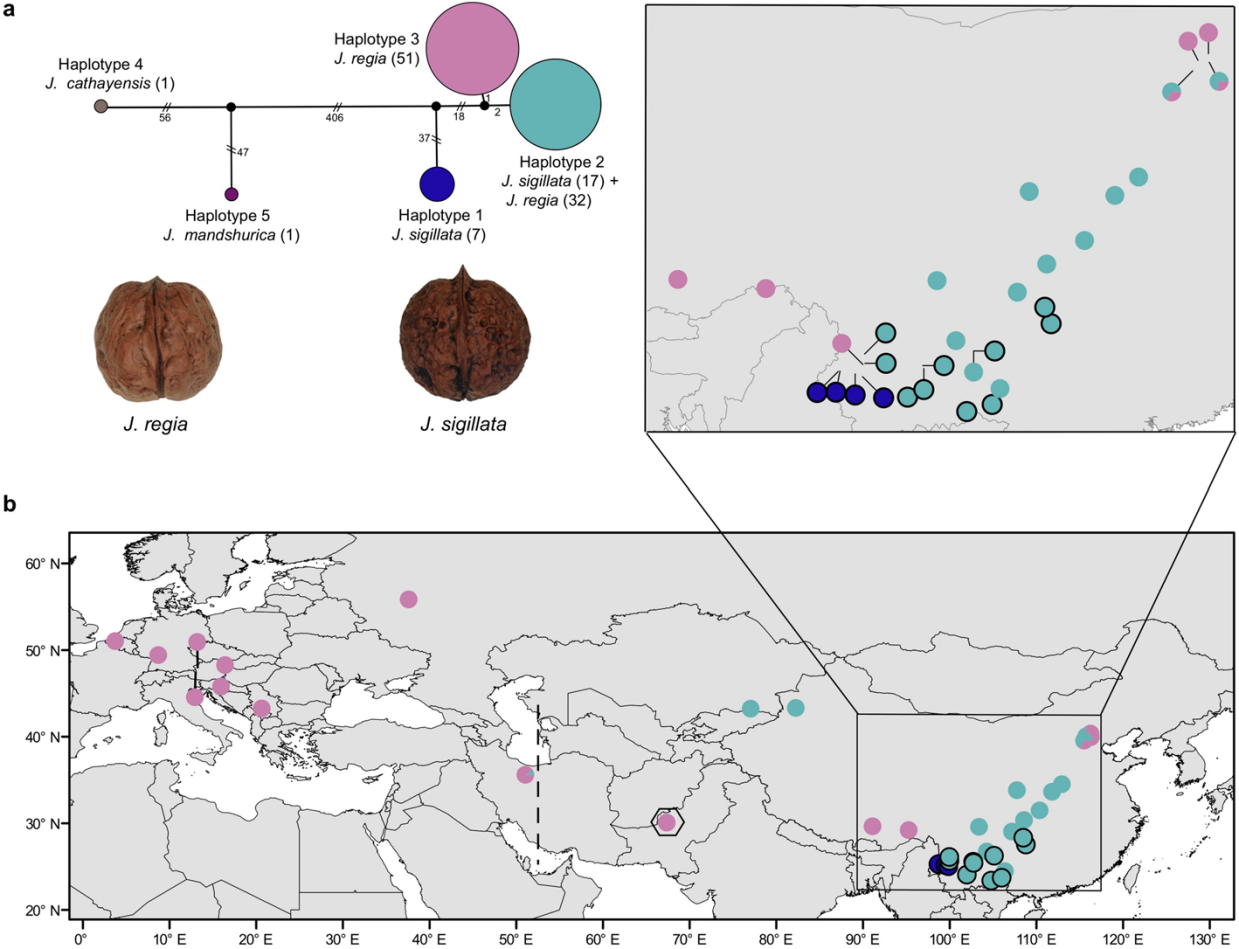
Network of the chloroplast DNA haplotypes present in *Juglans regia* and *J. sigillata.***a** Haplotype network based on whole-chloroplast genome variants. Numbers at branches are the number of mutations, the numbers in parentheses represent the number of individuals per haplotype, and circle diameters are proportional to the number of samples per haplotype. **b** Map showing the distribution of three chloroplast haplotypes in populations. Dots without black borders represent *J.regia*; dots with black borders represent *J. sigillata*. The hexagon marks walnut samples from Pakistan that were cultivated in Iran. The dashed line represents the separation between the western and eastern *J. regia* groups found in this study and highlights the sparse sampling in this crucial region

An ML tree obtained from whole-chloroplast genome data, showed three clades, with seven *J. sigillata* plastomes in one clade, another 17 *J. sigillata* and 32 *J. regia* plastomes in a second, and 51 *J. regia* plastomes in a third (Additional file 3: Fig. S4). A chloroplast haplotype analysis revealed only three haplotypes in the 107 trees from Belgium to China (the plastomes of the remaining 6 individuals had >1000 missing sites and were excluded from analysis, Additional file 2: Table S2). Haplotype 1 was unique to seven individuals of *J. sigillata*, haplotype 2 was shared by 32 *J. regia* and 17 *J. sigillata* individuals, and haplotype 3 was unique to *J. regia* (Fig. 4a). Haplotype 1 differs from haplotype 2 in 57 substitutions and from haplotype 3 in 56 substitions, whereas haplotype 2 differs from haplotype 3 in only three substitutions. That *J. sigillata* did not share a chloroplast haplotype with any western individuals is consistent with the scenario of recent gene flow from only eastern *J. regia* into *J. sigillata* (Fig. 3a).

## Discussion

### Geography and timeframe of the domestication of Persian walnut

Our results reveal two main genetic clusters of Persian walnuts, one including most (8/10) individuals from Iran and all individuals from Europe (Belgium to Serbia and Moscow), the other including the trees from China. This is consistent with other studies that found a genetic differentiation between eastern and western genotypes in walnut [52, 53]. The phylogeny obtained from the 22,048 whole-genome independent SNPs found in the 113 trees shows individuals from South Asia (Pakistan), West Asia (Iran), and China (Xinjiang in northwest-most China) in a basal position within the western *J. regia* clade, and one individual from Central Asia (Kazakhstan) in a basal position within the eastern *J. regia* clade. These results support western Central Asia as the geographic region of early walnut domestication [26, 41]. However, great caution should be exercised because our sampling in Central and West Asia is limited. Further work should sample this area more densely.

Regarding the question of bottlenecks during walnut domestication, our results using the GONE approach [23] reveal that the population sizes of western and eastern *J. regia* have been decreasing dramatically for the past 80 generations, that is, since ~2400 years ago (assuming a generation time of 30 years) or ~4000 years ago (assuming a generation time of 50 years; Methods). *Juglans regia* today has only two nearly-identical chloroplast haplotypes (one of which is shared with *J. sigillata* due to introgression), consistent with a serious bottleneck caused by human selection. Although wild *J. regia* trees occur in western China and in the Mediterranean region, people apparently planted the preferred, cultivated varieties passed on from Persia to the Greeks and the Romans (the Romans also were present in Iran around 113 AD), who then brought walnuts to all climatically suitable parts of Europe. Domesticated walnuts probably reached China from Turkestan, the region extending from Central Asia to Xinjiang, and thence spread toward today’s Gansu, Shaanxi, and more eastern provinces of China.

Our results from population genetics thus match archaeological finds that suggest that Persian walnuts have been gathered and apparently traded by humans from the Late Neolithic onwards, judging from nut remains found in ceramic containers in an Armenian grave dating to ~6200 years ago [43], nutshells from Kashmir dated to ~4700–4000 years ago [44], and nutshells in a former market site from Pakistan dated to ~3200 years ago [45], and fully domesticated forms appear to have existed between ~4000 years (Middle Bronze Age) and ~2400 years ago (during the Classic era), which would have been before the principal functioning of the Silk Road from about ~100 AD onwards [46].

### The domestication and evolutionary history of iron walnut, *J. sigillata*

Our results based on the IMa3 model [48] indicate that *J. regia* and *J. sigillata* diverged from each other by about 0.85 Ma, during the mid-Pleistocene, and the demographic models imply that both species’ population sizes have been decreasing dramatically for the past 1.0 Ma, with distinct trajectories since 0.4 Ma. A plausible explanation is that habitat fragmentation caused by climate oscillations during the Pleistocene may have fostered the divergence of *J. regia* and *J. sigillata* in southwest China. *Juglans sigillata* today contains only two chloroplast haplotypes (one of them shared with *J. regia*), and its population size has further decreased since ~600 years ago (assuming a generation time of 30 years) or 1000 years ago (assuming 50 years, which may be more likely given its flowering and fruiting age of 20+ years), implying a second bottleneck at this time.

Once domesticated forms of *J. regia* were introduced and cultivated in southwestern China (probably from Turkestan; above), the two species had a chance to meet and hybridize, and our results imply that the direction of gene flow was mainly from *J. regia* to *J. sigillata*, in agreement with earlier studies [54–56]. The gene tree of the shell-thickness gene (Fig. 3b) shows that *J. sigillata* has no private allele and is nested within the *J. regia* clade, indicating that this gene, which would have been important for human farmers, was introgressed from *J. regia* into *J. sigillata*. Different from the domestication of apples, pears, peaches, grapes, and apricots in which hybridization with wild relatives played an important role [11, 13–16, 18], hybridization played no role in the domestication of *J. regia*, while it was pivotal to the initial domestication of *J. sigillata*.

The extremely low plastome haplotype diversity in *J. regia*, with just two haplotypes that differ in three substitutions along their entire length of 135,640 bp, and only one unique haplotype in *J. sigillata*, suggests that both species comprise domesticated and feral individuals (because wild individuals would contain more diverse haplotypes). However, we sampled *J. sigillata* only in Yunnan and Guizhou, but not in the eastern Himalayas (southeast Tibet, Bhutan, and Sikkim), where it was, we hypothesize, probably first domesticated via hybridization with *J. regia*.

### Domestication bottlenecks are not an obsolete concept

Studies over the last three years, using computational approaches, especially the SMC method [20, 21], coupled with whole-genome data of domesticated species and their wild relatives, have often revealed a gradual decline in *N*_*e*_ over time, rather than the sudden population bottlenecks expected to occur with rapid domestication [4, 8, 10, 17, 57]. This has led to the suggestion that domestication is a protracted process and that selection intensity from unconscious (“natural”) selection may have been weak. However, our GONE results for *J. regia* and *J. sigillata* imply sudden population bottlenecks, rather than a protracted process, suggesting that the bottleneck scenario may well apply in at least some domesticated species.

However, there may also be a methodological bias. As Gaut et al. [10] (also see [23]) have pointed out, SMC methods tend to spread out bottlenecks over time, thereby lengthening the apparent domestication process. GONE appears less affected by this problem and more able to detect recent population bottlenecks [23]. Also, a population not exhibiting reduced heterozygosity does not preclude it having undergone a recent bottleneck [58]. Thus, it may be premature to abandon the concept of domestication bottlenecks.

## Conclusions

Our results support earlier suggestions that *J. regia* cultivation originated in western Central Asia and afterwards spread east to China and west to Europe, with a newly inferred bottleneck ~40 to 80 generations ago. By contrast, *J. sigillata* experienced a more recent bottleneck at ~20 generations ago when introgression from introduced *J. regia* (including of a gene resulting in thinner nutshells) may have facilitated its domestication. Hybridization thus played a significant role in iron walnut domestication. The inferred population bottlenecks during the domestication of both walnut species suggest that the bottleneck scenario may well apply in at least some perennial crop species.

## Methods

### Sampling and sequencing

We collected 52 mature *J. regia* individuals from Europe (28) and Asia (24), and 26 mature *J. sigillata* individuals from southern China (Fig. 4b). Genomic DNA was extracted from dried leaf tissue using a plant total genomic DNA kit (Tiangen, Beijing, China) and was then sequenced using paired-end libraries with an insert size of 350 bp on Illumina HiSeq X-ten instruments by NovoGene (Beijing, China), with read lengths of 150 bp. Samples were sequenced to an average depth of 30×. Additionally, we downloaded whole-genome resequencing data of *J. regia* from Iran (10 individuals) and Pakistan (8) and *J. sigillata* from southern China (9) from Ji et al. [29]; these data have an average depth higher than 17×. We also used genome resequencing data of *J. regia* (35) from northern China and *J. sigillata* (5) from southern China from our own previous studies, Zhang et al. [31] and Zhang et al. [59] (Additional file 2: Table S2).

To keep the samples genealogically independent, we filtered out related individuals using King v.2.2.7 [60]. If the kinship coefficient between a pair of individuals was larger than 0.0442 (corresponding to 3rd-degree relationship), we kept only the one that had a higher sequencing depth. In this way, 18 individuals of *J. regia* from Pakistan (3), Europe (5), and China (10), and 14 individuals of *J. sigillata* were removed from subsequent analysis (Additional file 3: Fig. S5). Among the remaining 113 individuals, any two are more distantly related to each other than 3rd-degree.

### Mapping and variant calling

Raw reads of the 113 individuals retained after kinship filtering were trimmed for adapters and low-quality reads using Trimmomatic v0.32 [61]. All clean reads were mapped to a *J. regia* reference genome [51] using BWA-MEM algorithm of BWA v.0.7.15 [62] with default settings. Only uniquely mapped and properly paired reads were used in the analyses. The SAMtools v.1.19 [63] were used to convert the Sequence Alignment Map to a Binary Alignment Map format file and to remove polymerase chain reaction duplicates. Subsequently, the SENTIEON DNAseq software package v. 202112 [64] was used to realign indels, call SNPs from each individual, and to joint SNPs from all individuals. To control the quality of genome-wide SNPs, sites with a mapping depth of less than a third or more than double of an individual’s average depth, nonbiallelic sites, and sites with missing data were removed. Next, heterozygous genotypes were called if the proportion of the nonreference allele was between 20% and 80% for a sequencing depth >20× [65] or if the proportion of the nonreference allele was between 10 and 90% for a sequencing depth >10×; otherwise, a homozygous genotype was called. After filtering, we obtained 6,792,732 SNPs. Then, to obtain neutral and independent SNPs, SNPs located in a coding sequence or its 20-kb extension region were discarded. In addition, the SNPs were thinned using a distance filter of interval >20 kb. Finally, singletons were excluded to reduce false-positive effects caused by sequencing error, resulting in a data set of 2352 SNPs for population structure analysis.

### Population structure

To investigate the population structure of the 113 individuals, a PCA was performed using the R package SNPRelate v. 1.6.2 [66] with default settings. STRUCTURE v. 2.3.4 [67] was used to cluster individuals based on *K* = 1–8, using the admixture model with correlated allele frequencies. To control for unequal sample sizes among species, we set POPALPHAS = 1 with an initial value of ALPHA = 0.25 as suggested by Meirmans [68], using 100,000 burn-in steps followed by 1,000,000 MCMC steps. Then, 20 runs were carried out for each cluster (*K*) to assess the degree of variation in the likelihood of each *K*. The optimal value of *K* was determined by Ln (D|K), the final posterior probability of *K* [67], and Delta *K*, the rate of change in Ln (D|K) between successive *K* values [69], and KFinder v1.0 according to the parsimony index (PI) of Wang [47]. Ultimately, 65 individuals of *J. regia* and 16 individuals of *J. sigillata* with an estimated posterior probability >0.80 at *K* = 2 were used for identifying the ghost introgression and detection of positive selection. We used 31, 25, and 15 individuals, respectively, from the western *J. regia*, eastern *J. regia*, and *J. sigillata* groups with an estimated posterior probability >0.80 at *K* = 3 for population demography and PHASE analysis.

### Phylogenetic analysis

To test whether the nine admixed individuals from Pakistan, Iran, Kazakhstan, and Xinjiang might represent a large ancestral large population, we constructed a phylogeny of the 113 individuals by using SVDquartets [70] with a total of 22,048 whole-genome SNPs that are at least 20-kbp apart from each other. We used VCFtools v0.1.17 [71] to extract the SNPs data of 31 individuals from the western group, 25 individuals from the eastern group, and the nine admixed individuals. We calculated the shared and private SNPs for the western and eastern group, as well as the nine admixed individuals*,* repeating the calculation 20×, each time with nine randomly selected individuals.

### Population demographic analysis

We used PSMC [20] to infer changes in *N*_*e*_ over time. As recommended, we used sequencing data with a mean genome coverage of ≥18, a per-site filter of ≥10 reads, and no more than 25% of missing data [72]. Four individuals of *J. regia* (western group), *J. regia* (eastern group), and *J. sigillata* were mapped to the *J. regia* reference genome [51]. The parameters in PSMC were set with quality adjusted to 50, the minimum mapping quality to 20, the minimum depth to one-third of average depth genome coverage, and maximum depth to 2-fold average depth genome coverage [73]. As in earlier studies [54, 73, 74], we assumed a walnut generation time of 30 or 50 years, and a recently inferred mutation rate of 1.54×10^−9^ per site per year (Ding et al., under review).

To infer changes in *N*_*e*_ in the recent past, we used GONE [23]. We assumed a constant rate of recombination of 2.63 cM/Mb for the whole genome [50] and excluded LD data with recombination rates >0.01 to reduce the effect of sampling on the estimates as well as artefacts from recent migrants from another population, following the GONE User’s Guide. We did 100 replicate analyses, each having 50,000 SNPs sampled randomly from each chromosome.

### Estimating divergence time and gene flow

To estimate the divergence time and gene flow among the walnut groups identified by STRUCTURE, we used Bayesian inference implemented in IMa3 v.1.11 [48]. IMa3 is a genealogy sampling program that implements a multi-population IM model with a “hidden genealogy” Markov-chain Monte Carlo (MCMC) update and allows for the inference of unsampled “ghost” populations to account for sampling gaps in the data set.

To obtain neutral and independent loci that IMa3 needs, we built consensus sequence of each individual. First, we mapped reads of each individual to *J. regia* reference genomes (JrSerr: http://aegilops.wheat.ucdavis.edu/Walnut/annotation/JrSerr_genome.fa) using the BWA-MEM algorithm from BWA v. 0.7.15. Second, we performed variant calling using SAMTOOLS v.1.19 [63] and filtered the SNPs with the quality adjuster -C setting to 50, the minimal mapping quality to 20. Indels or any SNPs within 3 bp around indels were removed. Then, a heterozygous genotype was called if the depth of a site was between 20× and the 2-fold average depth of each individual genome and the proportion of a nonreference allele was between 20 and 80%, or if the depth was between 6 and 20× and the proportion was 10–90%; otherwise a homozygous genotype was called [65]. Sites with mapping depths less than one-third of average depth or more than 2-fold average depth were masked as missing data in consensus sequence. To ensure neutrality, those SNPs located in a coding sequence or its 20-kb extension region were masked as missing data. A python script (https://github.com/Yamei-Ding/Juglans/tree/master/Demographic/IMa3/get_loci.py) was used to obtain 500–1000 bp long loci at 20-kb intervals on the genome that contain no missing data. Doing so results in a total of 184 loci for subsequent use in IMa3. For each locus, we reconstructed haplotypes using PHASE 2.1 [75], which implements a Bayesian statistical method for reconstructing haplotypes from population genotype data, and randomly selected 10 individuals (20 alleles) from each group to perform IMa3 analysis.

We compared both a ghost model (((western *J. regia*, eastern *J. regia*), *J. sigillata*), ghost population) and a non-ghost model ((western *J. regia*, eastern *J. regia*), *J. sigillata*). The parameters of splitting time, effective population size, and gene flow were estimated under both models. Two independent runs were carried out, and each run was conducted using a geometric heating scheme and with a chain length of 400. The HKY model of nucleotide substitution was used for all loci [76]. The substitution rate was set to 1.54×10^−9^ per year per site for all loci (Ding et al., under review). One genealogy was saved every 100 steps, with a 30-h burn-in prior to sampling, and at least 10,000 sampled genealogies. Marginal likelihood values for the ghost and non-ghost models were compared to decide which of them better fit our data. For both the non-ghost and ghost model, generation times were assumed to be 50 or 30 years.

### Ghost introgression and selective sweep

We used Sprime [49] to detect the segments of archaic introgression in *J. sigillata* with the information of the genetic distance in centimorgans for each SNP. Based on 6,792,732 high-quality SNPs, population-scaled recombination rates (*ρ* = 4*N*_e_*r*) were estimated using the LDhat v.2.2 [77, 78], with 10,000,000 MCMC iterations, sampling every 2000 iterations, a block penalty parameter of five, and a burn-in of the first 1,000,000 iterations when summarizing the results. We used the Kosambi formula [79]:

$$M=\frac{1}{4}\times \ln \frac{1+2r}{1-2r}$$

to calculate the genetic distance in centimorgans (*M*) for each SNP by recombination rates (*r*). In Sprime, we used *J. regia* as the outgroup to detect the segments of ghost introgression of *J. sigillata* with an assumed mutation rate of 7.7×10^−8^ per site per generation (generation time was 50 years) (Ding et al., under review) and a minscore of 1.5×10^5^ following Browning et al. [49].

We used five statistics from three methods to detect signatures of selection in *J. sigillata* as follows: Linkage disequilibrium-based cross-population extended haplotype homozygosity (XP-EHH) [80]; site-frequency-spectrum-based nucleotide diversity [81]; Tajima’s *D* [82], the composite-likelihood-ratio test (CLR) [83]; and the population differentiation-based *F*_*ST*_ [84]. Tajima’s *D*, *π* and *F*_*ST*_ were calculated by using VCFtools v0.1.17 [71] with 20-kb stepping windows. For each window, we computed Δπ as π_*J. regia*_/π_*J. sigillata*_. We performed the CLR test in 20-kb stepping windows using SWEEPFINDER2 [85] with the default parameter settings to scan for selective sweeps with pre-computed empirical spectrum and recombination map for *J. regia* and *J. sigillata*, respectively. We calculated XP-EHH using REHH v.3.2.2 R package [86] to find selective sweep regions in *J. sigillata*, assuming *J. regia* as neutral population. The identification of candidate regions under positive selection across the genome of *J. sigillata* was accomplished by combining information for the aforementioned five statistics in a single score using the decorrelated composite of multiple signals method (DCMS) [87].

### A phylogeny of the *Juglans* shell-thickness gene sequences

To annotate genes of interest, we used BLASTP (*E* ≤ 10^−10^) [88] to find the best match of protein sequences in the Swiss-Prot database, and to find genes related to fruit traits, we downloaded two sets of protein sequences for *J. regia*, Chandler v2.0 [50] and JrSerr [51]. BLASTP [88] (*E* < 10^−10^) was used to search for potentially homologous pairs of protein sequences between Chandler and JrSerr. The shell-thickness gene in Chandler is “Jr02_19210” and that in JrSerr is “Jr6DG00151300.” We extracted “Jr6DG00151300” from consensus genomes of *J. sigillata* and *J. regia,* and then used PHASE 2.1 [75] to do phasing. As Stephens et al. [89] pointed out, departures from the Hardy-Weinberg equilibrium in the data have little effect on the accuracy of their method, so 112 individuals (one individual was removed because of too many missing data) were put together for phasing. The constant model was employed for recombination rate variation, and MCMC iterations were set to 10,000 iterations (burn-in = 10,000; thinning interval = 10). When the phase certainty was set to 0.9 (*p* = *q* = 0.9), 74 individuals of *J. regia* and 20 individuals of *J. sigillata* were successfully phased, resulting in a total of 188 haploid sequences. The haplotypes of the 188 sequences were identified by DnaSP v6 [90] and then used with IQTREE [91] to construct a maximum-likelihood (ML) phylogeny for haplotypes of the shell-thickness gene.

### Chloroplast genome analysis

Reads from the 113 individuals were mapped to the *J. regia* chloroplast genome (NC_ 028617.1) using the BWA-MEM algorithm of BWA v.0.7.15. We then performed variant calling using SAMTOOLS v.1.19, with SNPs converted to the Variant Call Format (VCF). We used coverage to differentiate plastid and nuclear sequences, and for each position in the reference chloroplast genome, bases were called if the coverage was greater than 10-fold the nuclear genome average read depth and if more than 90% of the reads agreed for either the reference or an alternate base. Any position not meeting these criteria was called as missing data, and InDels were excluded from all analyses. If an individual genome data set was missing >1000 bp, it was removed from subsequent analysis. We downloaded *J. cathayensis* (NC_033893.1) and *J. mandshurica* (NC_033892.1) whole-chloroplast sequences as outgroups. After removing one inverted repeat region, all the sequences were aligned with MAFFT v.7.475 [92], and IQ-TREE v.2.1.2 [91] was used to build a ML gene tree using the ultrafast bootstrap approach (-B 1000). The haplotypes of all individuals were identified by DnaSP v 6[90], and a haplotype network was generated by PopArt version 1.7 (http://popart.otago.ac.nz).

## Supplementary Information

Additional file 1. Table S1. Three methods used to determine the optimal number of clusters in the *Juglans* STRUCTURE analyses.

Additional file 2. Table S2. Details of the sample locations, Q values at K=3 in STRUCTURE, and chloroplast haplotypes.

Additional file 3. Figure S1. Population-demographic history of *Juglans sigillata* and eastern and western *J. regia*. Figure S2. Divergence time and gene flow for a non-ghost model in IMa3 with generation times of 50 years. Figure S3. The distribution of ghost introgression segments identified with Sprime on chromosomes of *J. sigillata*. Figure S4. Plastid phylogeny for 24 individuals of *J. sigillata*, 83 of *J. regia*, one *J. mandshurica*, and one *J. cathayensis*, the latter two as outgroups. Figure S5. Relationships among 145 individuals of *J. regia* and *J. sigillata* inferred using Kinship-based INference for Genome-wide association studies (*KING*).

Additional file 4. Table S3. Parameter estimation of ghost models in IMa3 with generation times of 50 and 30 years.

Additional file 5. Table S4. Parameter estimation of non-ghost models in IMa3 assuming *Juglans regia* and *J. sigillata* generation times of 50 and 30 years.

Additional file 6. Table S5. Annotations of the six genes involved in biotic and abiotic stress responses.

Additional file 7. Table S6. Information on the haploid sequences of each haplotype of the shell-thickness gene.

Additional file 8. Review history.

## Data Availability

The entire genome resequencing data have been deposited at GenBank under the accession PRJNA356989 [94]. The custom scripts used in this study have been deposited in Github under MIT license (https://github.com/Yamei-Ding/Juglans) [95] and in Zenodo (https://zenodo.org/record/6736418) [96]. The whole-genome resequencing data of *J. regia* from Iran (10 individuals) and Pakistan (8) and *J. sigillata* from southern China (9) was from Ji et al. [29]; We also downloaded the genome resequencing data of *J. regia* (35) from northern China and *J. sigillata* (5) from southern China from our own previous studies [31, 59] (Additional file 2: Table S2). The *J. regia* reference genome was from Zhu et al. [51].
