# Supplementary material for: Population-genomic analyses reveal bottlenecks and asymmetric introgression from Persian into iron walnut during domestication
Source: Genome Biol. 2022 Jul 4;23:145. doi: 10.1186/s13059-022-02720-z (PMC9254524; doi:10.1186/s13059-022-02720-z)
Supplement: Supplementary file 3 — Additional file 3. Figure S1. Population-demographic history of Juglans sigillata and eastern and western J. regia. Figure S2. Divergence time and gene flow for a non-ghost model in IMa3 with generation times of 50 years. Figure S3. The distribution of ghost introgression segments identified with Sprime on chromosomes of J. sigillata. Figure S4. Plastid phylogeny for 24 individuals of J. sigillata, 83 of J. regia, one J. mandshurica, and one J. cathayensis, the latter two as outgroups. Figure S5. Relationships among 145 individuals of J. regia and J. sigillata inferred using Kinship-based INference for Genome-wide association studies (KING). [file 13059_2022_2720_MOESM3_ESM.docx]

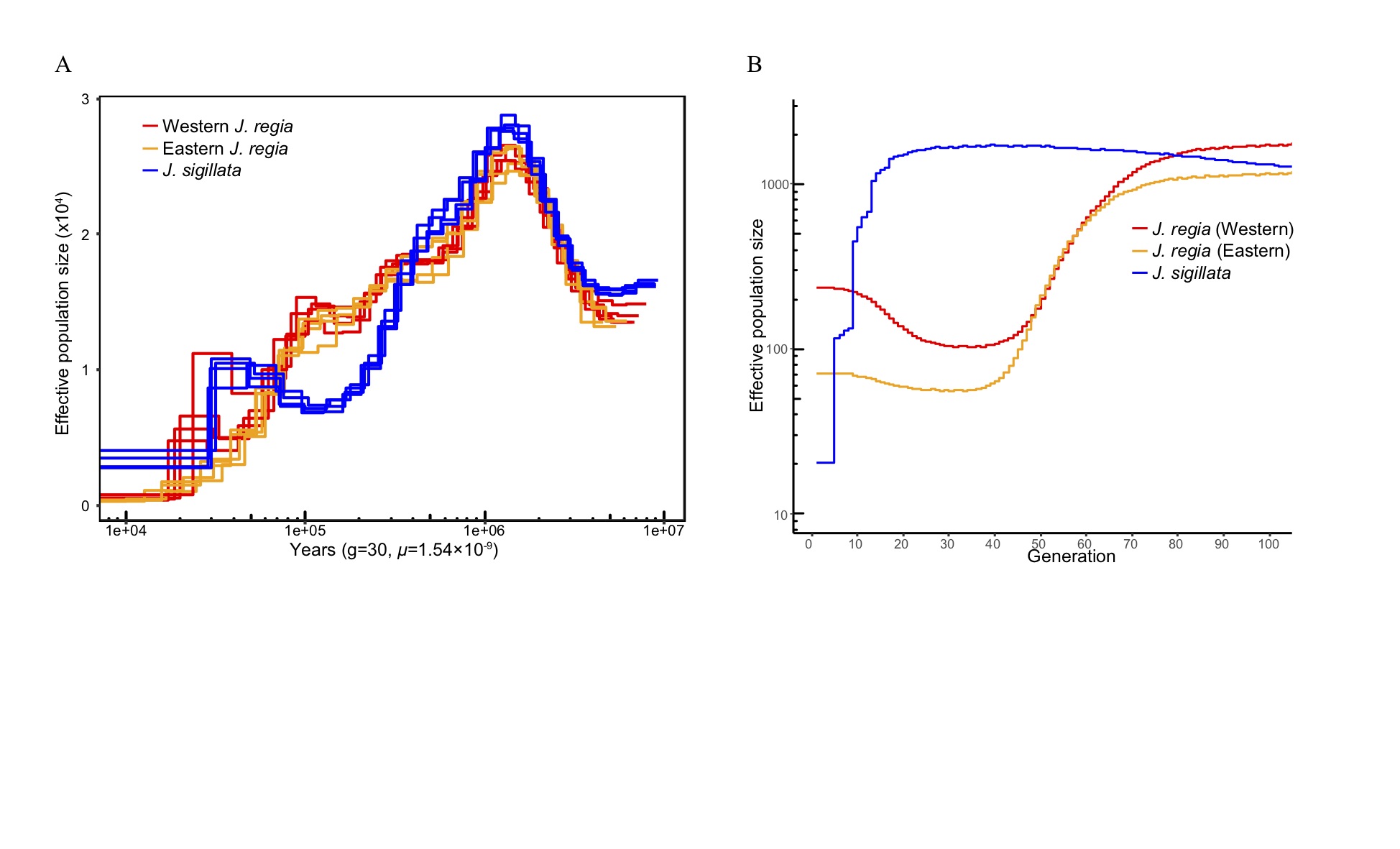
**Figure S1.** Population-demographic history of *Juglans sigillata* and eastern and western *J. regia*. Inferred by PSMC with a generation time of 30 years.


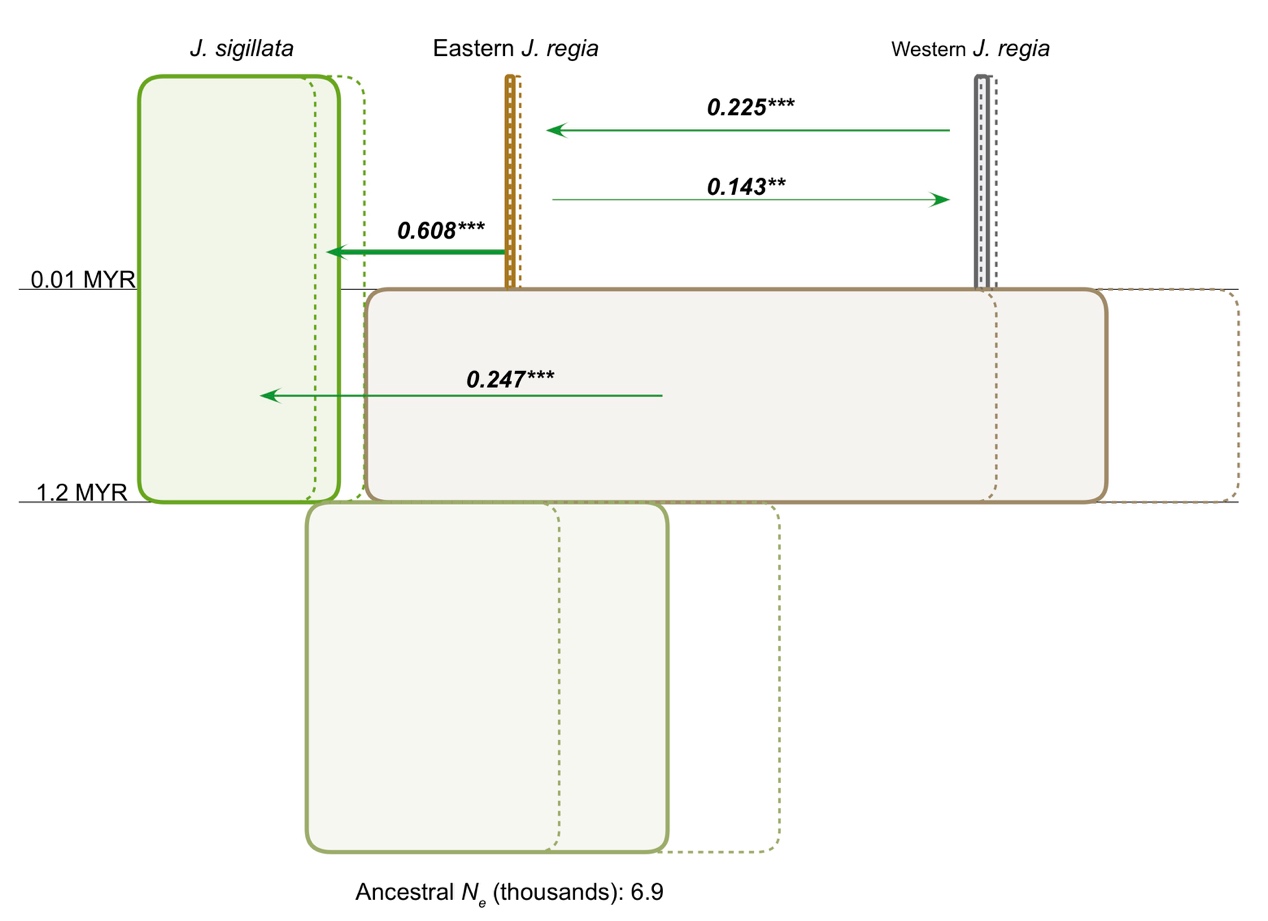


Figure S2. Divergence time and gene flow for a non-ghost model in IMa3 with generation times of 50 years. Each group is represented by a box, the width of which represents its effective population size (ancestral *N_e_* is given for scale). Confidence intervals are indicated as dashed-line boxes. Green arrows represent effective number of migrant gene copies per generation (*2Nm*) from the source population to the receiving population. Only statistically significant migration rates are shown (**P* < 0.05; ***P* < 0.01; ****P* < 0.001).

**
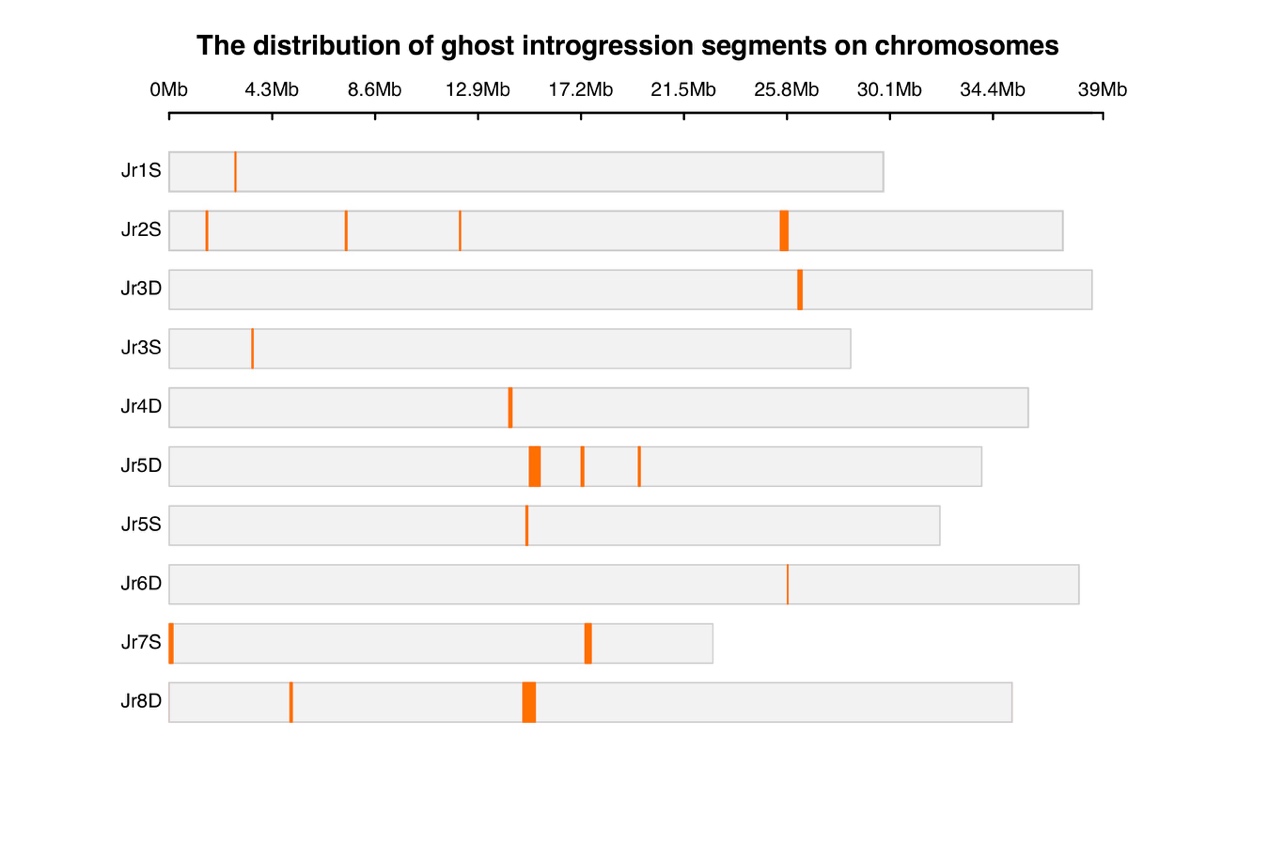
Figure S3.** The distribution of ghost introgression segments identified with Sprime on chromosomes of *J. sigillata*. The gray blocks stand for chromosomes and orange blocks stand for ghost introgression segments.


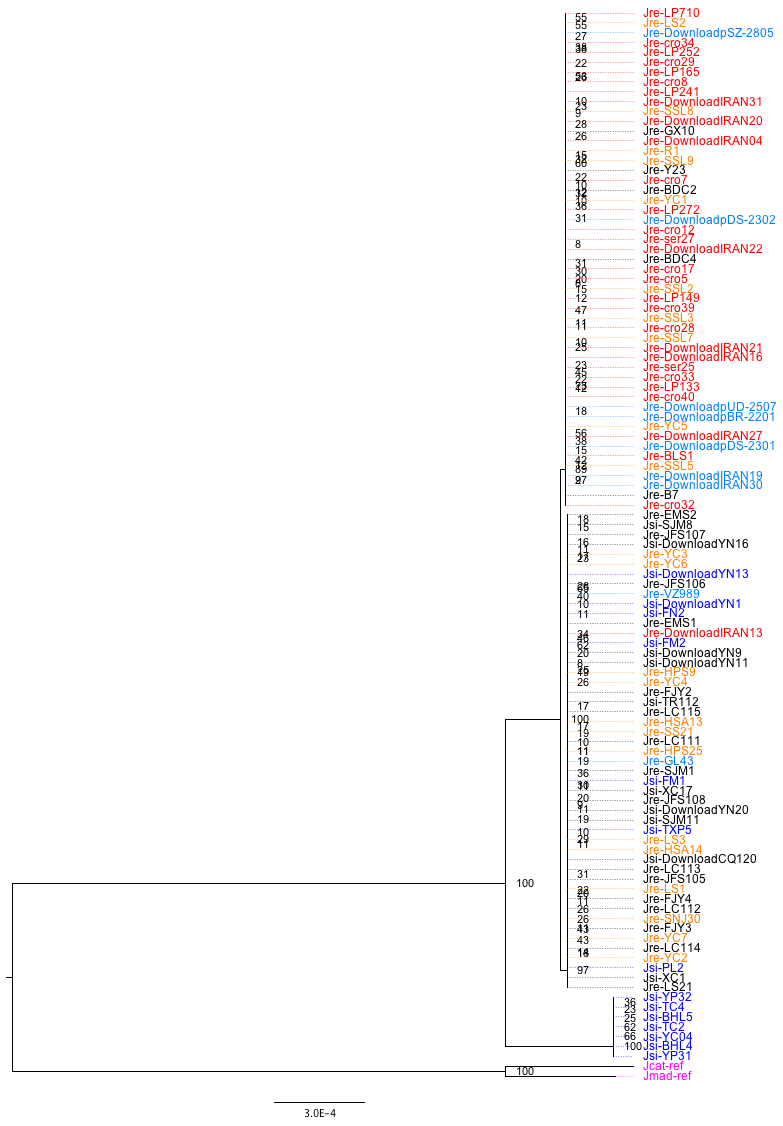
 Figure S4. Plastid phylogeny for 24 individuals of *J. sigillata*, 83 of *J. regia*, one *J. mandshurica*, and one *J. cathayensis*, the latter two as outgroups. Red and orange represent the western and eastern group of *J. regia*; blue represents *J. sigillata*; cyan represents hybrids between western and eastern *J. regia*; black represents hybrids between eastern *J. regia* and *J. sigillata*; and purple represents the outgroups. Numbers at nodes are ultrafast bootstrap (UFBoot) support values (%).


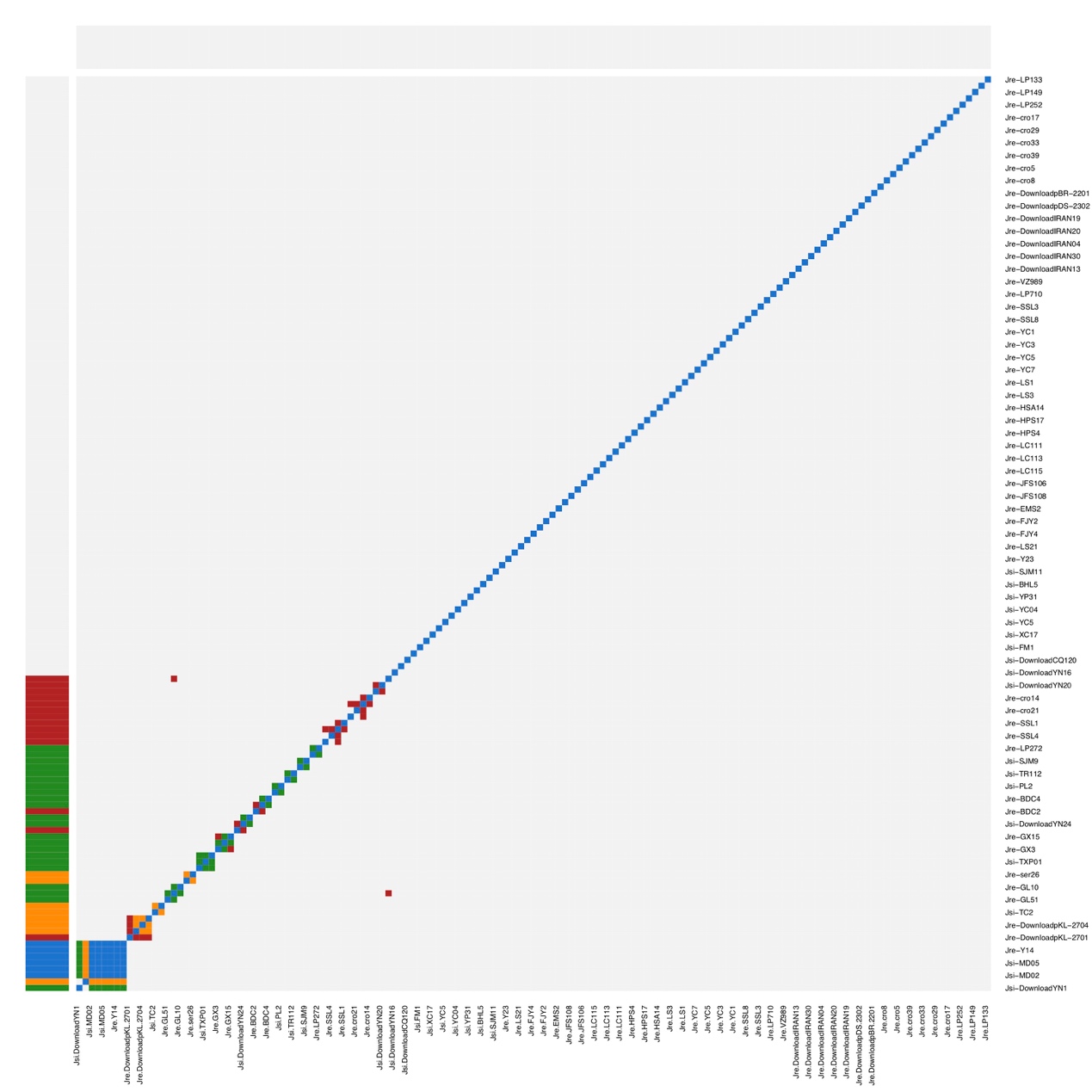


Figure S5. Relationships among 145 individuals of *J. regia* and *J. sigillata* inferred using Kinship-based inference for Genome-wide association studies (KING). Blue squares indicate duplicate/monozygotic (MZ) twin, orange squares indicate 1st-degree, green squares indicate 2nd-degree, and red squares indicate 3rd-degree relationships.
