# Supplementary material for: Population-genomic analyses reveal bottlenecks and asymmetric introgression from Persian into iron walnut during domestication
Source: Genome Biol. 2022 Jul 4;23:145. doi: 10.1186/s13059-022-02720-z (PMC9254524; doi:10.1186/s13059-022-02720-z)
Supplement: Supplementary file 8 — Additional file 8. Review history. [file 13059_2022_2720_MOESM8_ESM.docx]

Review History

**First round of review**

**Reviewer 1**

**Were you able to assess all statistics in the manuscript, including the appropriateness of statistical tests used?**

There are no statistics in the manuscript.

**Comments to author:**

The authors in this study show a useful insight into genome diversity in walnut through structure based analyses. The team convincingly show admixture between sigillata and regia associated with the east and give intriguing insight into the origins of walnut domestication. However, I find this paper somewhat distracted, and the emphasis currently sits on the weakest evidence. I would recommend at the very least a major rewrite and refocus.

The team then use an alternative to the classic MSMC approach recently published based on LD to calculate past population size on the basis of its robustness over recent timeframes and complex structures. I would be more cautious about the inferences here. The MSMC approach (which they also use) is well known to produce an artefactual pattern of decreasing population size over long periods of time when the sample is a substructured metapopulation, and indeed the teams results indicate this is likely to be the case for walnut in Fig 2a. The approach of Santiago has much to commend it, but under conditions of a metapopulation and low migration rate, it too suffers similar artefacts (see Fig 2. Panel F of Santiago et al 2020). I think the authors need to take this into account in their interpretation. I am disinclined to take this result at face value without some further model support or corroboration here.

The IMa3 analysis looks good, as does the evidence for introgressive movement into sigillata using Sprime. The authors make a convincing case that sigillata domestication is associated with regia introgression, with donation of shell-thickness alleles and chloroplast haplotypes. This is in itself very useful, and really makes the body of the study.

The emphasis on bottlenecks is at best weakly supported (I am not averse to walnuts having a bottleneck, seems very sensible to me). I get the sense the authors have been a little distracted by this 'hook'. This is particularly clear with their last few sentences which make a banal point that is really unrelated to their work, or, I think, the discussion on the prevalence of bottlenecks in domestication. The authors think they observe a bottleneck, but their parting observation is that a census size can be reduced temporarily without having an impact on genetic diversity. This is an obvious point, not what they observe, and not as far as I am aware contested anywhere. What's the point? It seems like a straw castle.

I would recommend that either the authors strengthen their bottleneck evidence (perhaps some simulation would help here), or refocus the paper on the evidence that is both strong and useful.

**Reviewer 2**

**Were you able to assess all statistics in the manuscript, including the appropriateness of statistical tests used?**

Yes, and I have assessed the statistics in my report.

**Comments to author:**

In this paper, the authors investigate domestication bottlenecks and demographic history of walnut. To do so, they rely on a dataset of 113 whole genome sequences from two species: J. regia from Europe and parts of Asia, and J. sigillata from Southern China. With these data, the authors inferred population structure and demographic history. Most of the analyses are fairly standard, although I was not familiar with the GONE method. The take-home of this analysis is that there is some evidence of introgression between the

This is a reasonable paper with competent analyses, about which I have only a few comments.

The first is about population bottlenecks. Rather than being under debate (as implied by the Abstract and the title of the last Discussion section), it is clear that they have occurred for many domesticated species, especially annuals (maize, rice, etc.). Much of the debate is centered, in the end, on semantics- i.e., is there just a population bottleneck or also introgression? Some authors (but not this group, thankfully!) don't seem to realize that the two phenomena are not mutually exclusive and that bottlenecks can occur over long timeframes. It's kind of strange that this paper mostly (but not entirely) cites the cases where they argue against them. Personally I would also change the title of the last section of the Discussion to being more conclusive: "Domestication bottlenecks are not an obsolete concept", but of course this is the authors prerogative.

The authors are right, though, in questioning the phenomenon of bottlenecks for perennials, for which there is little evidence so far, not only for the crops on line 48 but also for grapes, which is in some ways the pre-eminent (at least economically) perennial (see Myles et al. PNAS, Zhou et al. PNAS and NP, also recent paper from Morgante's group in Nat Comm). I found it curious that the reviews that focus especially on perennial domestication were not cited - e.g., see papers by Myles, Migicovsky, a 2015 review by Gaut, Diez and Morrell, and especially the review by Miller and Gross from 2011. It's hard to imagine a perennial domestication paper without citing at least some of these (with apologies if I missed the citations). Anyway, in the context of perennials, it is quite interesting that there is some evidence for bottlenecks in walnut.

I was puzzled by the very few SNPs that were used (2,352) for structure analyses. I realize these were filtered by various criterion, but the reduction from 6 million snps to 2,352 feels overly severe. I honestly suspect that it won't make much difference for structure, except perhaps it could lead to too few groupings (although likely not incorrect groupings).

The GONE analyses are really interesting, because they do suggest bottlenecks, but quite a bit after (I think?) the time-scale of archaeological evidence for the use of Persian walnut. That is an interesting and fun inference, and I believe the explanation in the Discussion (replacement of genotypes after human migration). It also makes the important point indirectly that archaeological evidence, while very powerful, has some important limitations, too (especially the availability of samples across space and time)

Stylistically, this paper is well written, because the prose is very clear and excellent. However, the Results section is quite abbreviated, to the extent that I found some of it hard to follow. Here's one example: the mention of ghost populations (line 176) needs to be explained. I realize it is explained in the Methods, but I believe it should be explained well enough (briefly) in the Results to be able to follow the results, the motivation for including a ghost population, etc. Another example is the introgression section… the Sprime results are potentially really interesting, but they are kind of ignored except in the context of sweeps. There is some really interesting literature on the property of introgressed regions, and I (for one) would like the know where these sections are within the genome, whether Sprime evidence is corroborated by other methods (phylogenetics, ABBA-BABA, etc.). In general, I thought the authors perhaps did themselves a disservice by not being more comprehensive in the explanation of their Results and, in some cases, diving further into the results.

**Authors Response**

**Point-by-point responses to the reviewers’ comments:**

**Reviewer 1**:

The authors in this study show a useful insight into genome diversity in walnut through structure-based analyses. The team convincingly show admixture between sigillata and regia associated with the east and give intriguing insight into the origins of walnut domestication. However, I find this paper somewhat distracted, and the emphasis currently sits on the weakest evidence. I would recommend at the very least a major rewrite and refocus.

*Response 1: In the revised version, we have deemphasized the domestication bottleneck by changing the title, reorganizing the Introduction, and rewording the description of bottleneck throughout the text.*

The team then use an alternative to the classic MSMC approach recently published based on LD to calculate past population size on the basis of its robustness over recent timeframes and complex structures. I would be more cautious about the inferences here. The MSMC approach (which they also use) is well known to produce an artefactual pattern of decreasing population size over long periods of time when the sample is a sub-structured metapopulation, and indeed the team’s results indicate this is likely to be the case for walnut in Fig 2a. The approach of Santiago has much to commend it, but under conditions of a metapopulation and low migration rate, it too suffers similar artefacts (see Fig 2. Panel F of Santiago et al 2020). I think the authors need to take this into account in their interpretation. I am disinclined to take this result at face value without some further model support or corroboration here.

*Response 2:* *Thanks for your comment. Santiago et al. (2020) indeed mention that if the population has recent migrants from another population, the estimation of N_e_ will be biased, and they proposed that this can be partly corrected by using a lower maximum value of c (hc = 0.01) than the one recommended, as explained in the GONE User’s Guide. In our revision, we now explain why we set hc = 0.01. (line 182-183; line 440-442). Further support for the Persian walnut experiencing a domestication bottleneck comes from the IMa3 results where the eastern and western Juglans regia lineages diverged from each other, along with a strong population decline at 2,639–15,963 years, matching the GONE inference of a population bottleneck beginning from about 80 generations onwards (which equals 4000 years assuming a generation time of 50 years or 2400 years assuming a generation time of 30 years). Although we think the evidence for a domestication bottleneck is decent, we toned down our statements about a walnut domestication bottleneck in response to the reviewer’s concern (see response 1).*

The IMa3 analysis looks good, as does the evidence for introgressive movement into sigillata using Sprime. The authors make a convincing case that sigillata domestication is associated with regia introgression, with donation of shell-thickness alleles and chloroplast haplotypes. This is in itself very useful, and really makes the body of the study.

*Response 3: Thanks for your comments.*

The emphasis on bottlenecks is at best weakly supported (I am not averse to walnuts having a bottleneck, seems very sensible to me). I get the sense the authors have been a little distracted by this 'hook'. This is particularly clear with their last few sentences which make a banal point that is really unrelated to their work, or, I think, the discussion on the prevalence of bottlenecks in domestication. The authors think they observe a bottleneck, but their parting observation is that a census size can be reduced temporarily without having an impact on genetic diversity. This is an obvious point, not what they observe, and not as far as I am aware contested anywhere. What's the point? It seems like a straw castle.

*Response 4:* *While it may be obvious that a population’s census size can be reduced temporarily without having an impact on genetic diversity,* *reduced genetic diversity has been used to infer demographic bottlenecks (e.g.* *Eyre-Walker et al. 1998, PNAS; Zhu et al. 2007, MBE; Meyer et al. 2016, Nature Genetics; Velasco et al. G3: Genes, Genomes, Genetics 2016; Spengler, Frontiers in Plant Science 2019; Allaby et al. Trends in Ecology & Evolution 2022). We reworded our sentences to make our point here clearer.*

I would recommend that either the authors strengthen their bottleneck evidence (perhaps some simulation would help here), or refocus the paper on the evidence that is both strong and useful.

*Response 5: We have followed this advice (see response 1).*

**Reviewer 2**: In this paper, the authors investigate domestication bottlenecks and demographic history of walnut. To do so, they rely on a dataset of 113 whole genome sequences from two species: J. regia from Europe and parts of Asia, and J. sigillata from Southern China. With these data, the authors inferred population structure and demographic history. Most of the analyses are fairly standard, although I was not familiar with the GONE method. The take-home of this analysis is that there is some evidence of introgression between the Persian walnut and Iron walnut. This is a reasonable paper with competent analyses, about which I have only a few comments.

The first is about population bottlenecks. Rather than being under debate (as implied by the Abstract and the title of the last Discussion section), it is clear that they have occurred for many domesticated species, especially annuals (maize, rice, etc.). Much of the debate is centered, in the end, on semantics- i.e., is there just a population bottleneck or also introgression? Some authors (but not this group, thankfully!) don't seem to realize that the two phenomena are not mutually exclusive and that bottlenecks can occur over long timeframes. It's kind of strange that this paper mostly (but not entirely) cites the cases where they argue against them. Personally, I would also change the title of the last section of the Discussion to being more conclusive: "Domestication bottlenecks are not an obsolete concept", but of course this is the authors prerogative.

*Response 6**:* *Thanks for your suggestion. We have added more citations to give a more balanced review in line 49 (**Eyre-Walker et al. 1998, PNAS; Zhu et al. 2007, MBE; Meyer et al. 2016, Nature Genetics; Wang et al. 2017, Genome Biology; Gaut et al. 2018, Nature Plants). We have followed your advice to change the title of the last section of the Discussion to "Domestication bottlenecks are not an obsolete concept."*

The authors are right, though, in questioning the phenomenon of bottlenecks for perennials, for which there is little evidence so far, not only for the crops in line 48 but also for grapes, which is in some ways the pre-eminent (at least economically) perennial (see Myles et al. PNAS, Zhou et al. PNAS and NP, also recent paper from Morgante's group in Nat Comm). I found it curious that the reviews that focus especially on perennial domestication were not cited - e.g., see papers by Myles, Migicovsky, a 2015 review by Gaut, Diez and Morrell, and especially the review by Miller and Gross from 2011. It's hard to imagine a perennial domestication paper without citing at least some of these (with apologies if I missed the citations). Anyway, in the context of perennials, it is quite interesting that there is some evidence for bottlenecks in walnut.

*Response 7: Many thanks for these suggestions**. We now cite more review articles focusing on domestication of perennial crops in line 57, 121 and 315.*

I was puzzled by the very few SNPs that were used (2,352) for structure analyses. I realize these were filtered by various criterion, but the reduction from 6 million snps to 2,352 feels overly severe. I honestly suspect that it won't make much difference for structure, except perhaps it could lead to too few groupings (although likely not incorrect groupings).

*Res*p*onse 8:* *STRUCTURE analysis requires SNPs to be neutral and unlinked. To best meet these requirements, we employed more stringent filtering strategies than usual. However, to address the reviewer’s concern of too fewer SNPs left after such strict filtering, we relaxed the distance-filtering criterion (from 20k to 5k) to obtain a dataset of 24,791 SNPs, increased by more than an order of magnitude, and then reanalysed population structure. As can be seen, the larger dataset produced almost identical grouping as the original, smaller dataset.*

*Fig. 1 The result of STRUCTURE analysis of 113 individuals using 2352 SNPs in the main text and 24791 SNPs with the new dataset.*

The GONE analyses are really interesting, because they do suggest bottlenecks, but quite a bit after (I think?) the time-scale of archaeological evidence for the use of Persian walnut. That is an interesting and fun inference, and I believe the explanation in the Discussion (replacement of genotypes after human migration). It also makes the important point indirectly that archaeological evidence, while very powerful, has some important limitations, too (especially the availability of samples across space and time).

*Response 9: Thank you for your comments.*

Stylistically, this paper is well written, because the prose is very clear and excellent. However, the Results section is quite abbreviated, to the extent that I found some of it hard to follow. Here's one example: the mention of ghost populations (line 176) needs to be explained. I realize it is explained in the Methods, but I believe it should be explained well enough (briefly) in the Results to be able to follow the results, the motivation for including a ghost population, etc. Another example is the introgression section… the Sprime results are potentially really interesting, but they are kind of ignored except in the context of sweeps. There is some really interesting literature on the property of introgressed regions, and I (for one) would like the know where these sections are within the genome, whether Sprime evidence is corroborated by other methods (phylogenetics, ABBA-BABA, etc.). In general, I thought the authors perhaps did themselves a disservice by not being more comprehensive in the explanation of their Results and, in some cases, diving further into the results.

*Response 10: Thanks for your suggestion. We have added more details in the Result (line 195-200; line 216-220; line 229-232). In the below, Fig. 2 gives the information on the distribution of ghost introgression sections identified using Sprime approach within the genome of J. sigillata and is as Fig. S3 in the supplementary.*

*Fig. 2.* *The distribution of ghost introgression segments (orange) identified with Sprime on chromosomes of J. sigillata*.

**Second round of review**

**Reviewer 1**

This revised version of the manuscript is improved. The authors do a better job of presenting the bottleneck evidence, although the authors should be aware that that it is still likely there is some artifactual bias - reducing the maximum recombination rate serves to ameliorate the artifactual signal (by considering fewer and closer loci), but will not remove it entirely. The language of the manuscript is suitably circumspect on this point. It does help that a corroborating signal comes through the IMa3 analysis. The inclusion of further perennial studies in the citations is to the good also. The discussion is toned down appropriately. I am happy for this to go out now.

**Reviewer 2**

I have reviewed the changes and find the revisions satisfactory.
